# Experimental and Theoretical Investigations of MAPbX_3_‐Based Perovskites (X=Cl, Br, I) for Photovoltaic Applications

**DOI:** 10.1002/open.202300055

**Published:** 2023-10-24

**Authors:** Sonali Mehra, Rahul Pandey, Jaya Madan, Rajnish Sharma, Lalit Goswami, Govind Gupta, Vidya Nand Singh, Avanish Kumar Srivastava, Shailesh Narain Sharma

**Affiliations:** ^1^ CSIR– National Physical Laboratory Dr K. S. Krishann Road New Delhi India 110012; ^2^ AcSIR– Academy of Scientific and Innovative Research Ghaziabad India 201002; ^3^ VLSI Centre of Excellence Chitkara University Institute of Engineering and Technology Chitkara University Punjab India; ^4^ CSIR– Advanced Materials and Processes Research Institute Bhopal Madhya Pradesh India 462026

**Keywords:** halide perovskites, hot injection, colloidal synthesis, simulation, SCAPS-1D

## Abstract

This work mainly focuses on synthesizing and evaluating the efficiency of methylammonium lead halide‐based perovskite (MAPbX_3_; X=Cl, Br, I) solar cells. We used the colloidal Hot‐injection method (HIM) to synthesize MAPbX_3_ (X=Cl, Br, I) perovskites using the specific precursors and organic solvents under ambient conditions. We studied the structural, morphological and optical properties of MAPbX_3_ perovskites using XRD, FESEM, TEM, UV‐Vis, PL and TRPL (time‐resolved photoluminescence) characterization techniques. The particle size and morphology of these perovskites vary with respect to the halide variation. The MAPbI_3_ perovskite possesses a low band gap and low carrier lifetime but delivers the highest PCE among other halide perovskite samples, making it a promising candidate for solar cell technology. To further enrich the investigations, the conversion efficiency of the MAPbX_3_ perovskites has been evaluated through extensive device simulations. Here, the optical constants, band gap energy and carrier lifetime of MAPbX_3_ were used for simulating three different perovskite solar cells, namely I, Cl or Br halide‐based perovskite solar cells. MAPbI_3_, MAPbBr_3_ and MAPbCl_3_ absorber layer‐based devices showed ~13.7 %, 6.9 % and 5.0 % conversion efficiency. The correlation between the experimental and SCAPS simulation data for HIM‐synthesized MAPBX_3_‐based perovskites has been reported for the first time.

## Graphical abstract

MAPbX_3_ (X=Cl/Br/I) perovskites have been prepared using the colloidal hot‐injection method (HIM). Their structural, morphological, and optical properties are characterized, and photovoltaic properties are studied using simulations. The MAPbI_3_ perovskite possesses a low band gap, low carrier lifetime but the highest PCE, making it a promising candidate for solar cell technology.

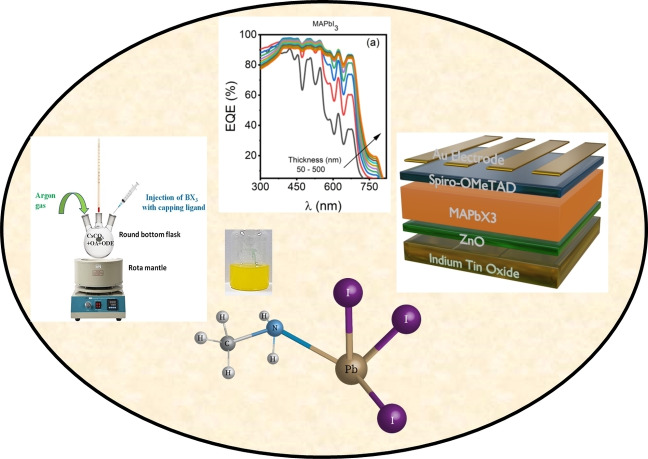

## Introduction

Hybrid organic‐inorganic methylammonium lead halide MAPbX_3_ (X=Cl, Br, I) perovskites have been known as a very efficient absorber layer in solar cells because of the various properties such as high absorption coefficient,^[1]^ high carrier mobility,^[2]^ tunable band gap^[3]^ and simple solution‐processed synthesis technique.^[4]^ Hybrid organic‐inorganic perovskite solar cell technology is an emerging field compared to conventional solar cells.[^5^, ^6^, ^7^] Perovskite solar cells possess low cost, ease of fabrication, high device performance[^8^, ^9^] and enhanced electrical and optical properties, allowing the absorption of near‐infrared light and the visible light spectrum. All these properties make the perovskite a better candidate than conventional silicon solar cells, which only absorb the visible‐light spectrum.^[10]^

**Figure 1:**
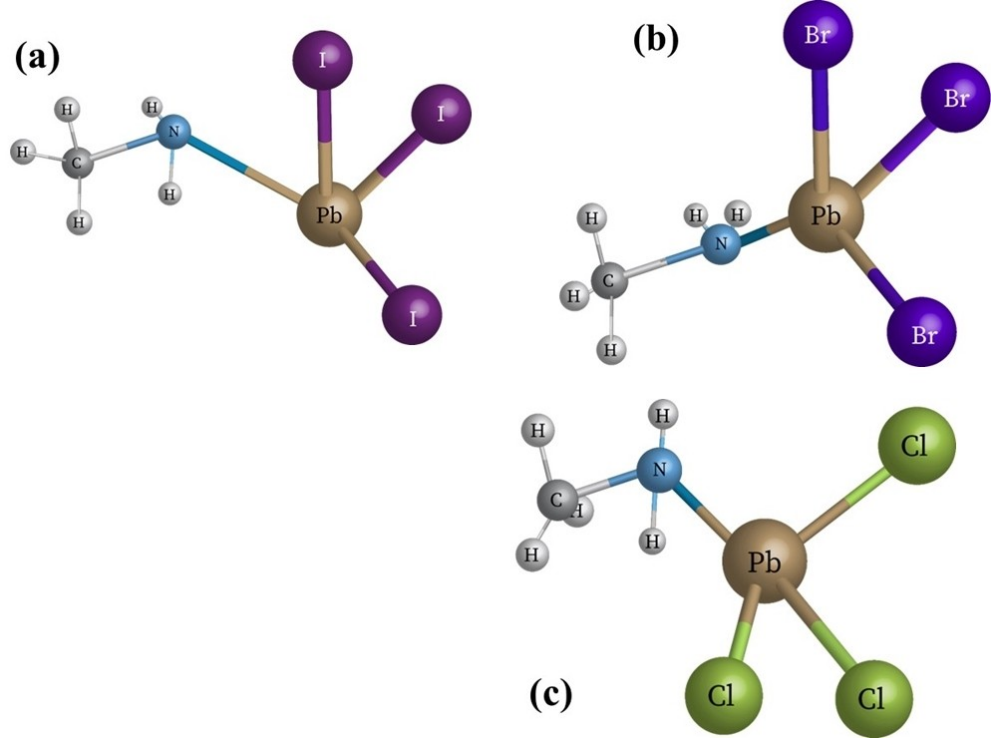
Chemical representation of (a) MAPbI_3_, (b) MAPbBr_3_, and (c) MAPbCl_3_.

Generally, perovskites possess the ABX_3_ structure where A is a large monovalent inorganic or organic cation (e. g., MA^+^, FA^+^, Cs^+^, etc.), B is a smaller inorganic divalent cation (e. g., Pb^2+^, Sn^2+^, Cu^2+^, Bi^2+^, etc.) and X is the halogen ion (X=Cl^−^, Br^−^, I^−^) which can bind with both A and B cations. The properties of perovskite materials can be reformed by altering the A, B or X site in the ABX_3_ perovskite lattice.^[11]^ Figure 1 presents the geometric 3D visualization of the perovskite structure used for calculating the dipole moment and binding energy between their atoms in chemical structures. They also help to determine the perovskite‘s capability to donate and accept electrons. As per general standards, the tolerance factor of perovskites should lie in the range of 0.8<T<1 to obtain a stable perovskite. T′s value is essential in maintaining and holding the bond among the cations.^[12]^ Specifically, for perovskite structure, cation A≫cation B; thus, the methylammonium (MA^+^) ion was considered as the promising organic material for perovskite solar cells.[^13^, ^14^]

Due to the unique optoelectronic properties of lead‐halide perovskite materials, they were widely used as absorber layers in solar cells.[^15^, ^16^] Their efficient emission characteristics make them a potential candidate for emitter devices.[^17^, ^18^] But still, the primary concern is to provide resistance to perovskites from degradation and replacement of expensive hole transport layers (HTL) with cost‐effective HTL materials because, as per reported literature, HTL material plays an essential role in organic photovoltaics and organic optoelectronics as they help to reduce the degradation in device performance.[^6^, ^19^]

Now, since 2009, these hybrid perovskite solar cells have shown a significant increase in PCE from 3.8 % to 25.6 %.[^20^, ^21^] A tremendous increment was observed in the PCE of solar cells in the last 7–8 years.[^22^, ^23^] The primary factor that leads to the sharp increase in the performance efficiency of photovoltaics within a short period is the hybrid concept where both inorganic and organic solar cells are utilized based on efficient operation principles.[^24^, ^25^] Secondly, relatively more straightforward fabrication techniques provided a vital pathway toward economic solar energy alternatives.[^26^, ^27^, ^28^] Thus, the perovskite solar cells have attained higher efficiency because of the possible optoelectronic properties such as tuneable band gap,[^29^, ^30^] high absorption coefficient over the solar spectrum,[^27^, ^31^] the low exciton binding energy (BE)[^32^, ^33^] and effective charge transport characteristics.[^34^, ^35^] Furthermore, depending on the above properties of photovoltaics, different device designs can be developed, resulting in the enhanced PCE of perovskite solar cells.[^8^, ^36^]

Nowadays, photovoltaic devices hold great promise in reducing fossil energy dependence. The hybrid organic‐inorganic MAPbX_3_ perovskites were broadly known due to their excellent semiconducting properties.^[37]^ However, MAPbI_3_ perovskite possesses a long charge carrier diffusion length^[38]^ leading to the low recombination efficiency of holes and electrons in this perovskite material.[^39^, ^40^] Recently, this material has played an essential role in improving the performance of inverted devices where the perovskite material MAPbI_3_ layer is sandwiched between organic electron and hole blocking layer.^[41]^ This inverted configuration leads to a stable and highly efficient device without hysteresis effects and results in optimized high PCE.^[42]^

This article reports the synthesis of crystalline MAPbX_3_ (X=Cl, Br, I) using PbX_2_ and MAX as the precursors and the capping ligands using the hot‐injection technique under controlled conditions. The structural, optical, and morphological properties were studied using X‐ray diffraction (XRD), Photoluminescence (PL), UV‐Vis, Field Emission‐Scanning electron microscopy (FE‐SEM) and Transmission electron microscopy (TEM), which gives us an idea about the crystallinity and topographical properties of the perovskite materials. However, the change in the band gap, absorption coefficient, carrier lifetime, emission wavelength, morphology and other properties with halide ion variation were considered significant factors for the simulation study of the perovskite material. The device design was carried out using the simulation tool SCAPS 1D. Thus, depending on the simulation parameters, the power conversion efficiency (PCE) of the solar cell device was evaluated regarding halide ion variation in the perovskite material.[^43^, ^44^] As per the author's knowledge, the given article is a first‐hand report about the correlation of experimental and simulation data for MAPbX_3_ (X=Cl, Br, I) perovskites synthesized via cost‐effective, non‐vacuum‐based Hot Injection method.

## Experimental Section

### Materials

All the chemicals required for synthesis such as lead(II) bromide (PbBr_2_, 99.99 % trace metals basis), lead(II) iodide (PbI_2_, 99.99 % trace metals basis), lead(II) chloride (PbCl_2_, 99.99 % trace metals basis), methylammonium iodide (CH_3_NH_2_⋅HI/MAI, ≥99 % anhydrous), methylammonium bromide (CH_3_NH_2_⋅HBr/MABr, ≥99 % anhydrous), methylammonium chloride (CH_3_NH_3_Cl/MACl, for synthesis), oleyl‐amine (OLA, 70 %), oleic acid (OA, 90 %), octadecene (ODE, 90 %) and tri‐octyl phosphine (TOP, 90 %) were purchased from Sigma Aldrich. All the chemicals were used without any further purification.

**Figure 2:**
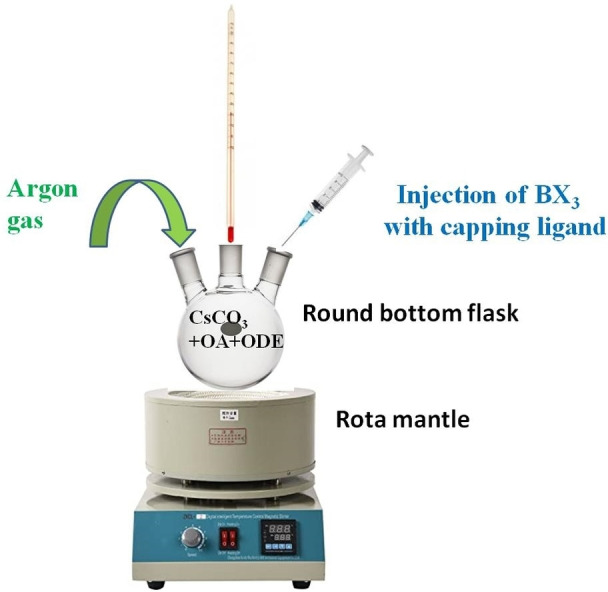
Diagrammatic representation of the Hot‐Injection technique.

In a typical synthesis, MAPbI_3_ was synthesized using the hot‐injection method and the diagrammatic representation of this method is shown in Figure 2. Here, 1 mM of MAI in 3 mL of OA was taken in 100 mL 3‐neck RBF with continuous stirring. This solution was heated to 100 °C until the clear solution was obtained under the Argon atmosphere for around 1hr. On the other hand, 0.5 mM PbI_2_ precursor dissolved in TOP was used as the injection solution for this synthesis. Then, the PbI_2_ solution was injected rapidly into the reaction solution under the argon atmosphere. After injection, the temperature was increased slowly to 195 °C for a specific time of 10 min. After 10 min, the heat source was removed, and the solution was cooled down to 60 °C under normal atmospheric conditions. Further, the solution obtained was centrifuged at 5500 rpm for 5 min, and discarded the supernatant. Later, it was rewashed with toluene and centrifuged at 8000 rpm for 7 min, and the supernatant thus obtained was utilized for characterization.

Similarly, MAPbBr_3_ and MAPbCl_3_ were also synthesized using the same hot‐injection technique. For MAPbBr_3_ synthesis, MABr and PbBr_2_ were used as the precursor salts, while MACl and PbCl_2_ were used as the precursors for the synthesis of MAPbCl_3_ perovskite. The synthesis conditions for all the halide perovskites are given below in Table 1., PbCl_2_ was dissolved in the stoichiometric ratio of ODE and OLA, whereas PbBr_2_ was dissolved in the solvent mixture OA and ODE.

**Table 1:** Reaction conditions for the synthesis of MAPbX_3_ (X=Cl, Br, I) perovskites.

Perovskite	Injecting solution	Temperature (°C)	Capping Ligands	Solvent	Atmosphere
MAPbI_3_	PbI_2_	190	TOP	Toluene	Argon
MAPbBr_3_	PbCl_2_	200	OA and ODE	Toluene	Argon
MAPbCl_3_	PbBr_2_	180	ODE and OLA	Toluene	Argon

Here, the addition of capping ligands is very crucial in the synthesis of halide‐based colloidal perovskites. Oleyl‐amine helps to control the crystallization process and gives a colloidal solution, and it will also help to stabilize the halide perovskites. However, without adding oleic acid in the synthesis, the colloidal solution becomes cloudy after storage for 24 h. Oleic acid also plays an essential role in suppressing the aggregation effects and helps to stabilize the halide perovskite.[^45^, ^46^] Moreover, toluene is used for washing the perovskites because its reaction kinetics is slow and leads to the destruction of colloidal particles during centrifugation due to the high sensitivity and instability of halide‐based perovskites.[^47^, ^48^]

### Characterization

XRD patterns of MAPbX_3_ (X=Cl/Br/I) perovskites were zanalyzed using a Rigaku X‐ray Diffractometer. The diffraction pattern was recorded using Cu Kα wavelength (λ=1.54056 Å) at a step rate of 0.02 min^−1^. The average particle size of perovskites was studied by Transmission Electron Microscopy (TEM), make and model JEOL JEM‐1011 (JEOL Ltd. Inc., Tokyo Japan) operated at 80–100 kV and the morphology of MAPbX_3_ (X=Cl, Br, I) perovskites was determined using Field Emission‐Scanning Electron Microscopy (FE‐SEM), model TESCAN MAGNA GMH. UV absorption spectra of halide perovskites were determined using Ocean Optics 400 tabletop UV‐Vis spectrophotometer. PL and TRPL studies of MAPbX_3_ (X=Cl, Br, I) halide perovskites were determined using an Edinburgh instrument equipped with a xenon lamp; model FLS 980 at different excitation and emission wavelength with respect to halide variation and TRPL was studied using the same instrument using the femtosecond laser. TRPL study helps to determine the average carrier lifetime required by the different halide perovskites. Thus, the supernatant material obtained after the above synthesis in the case of all the perovskites was utilized in the form of liquid or thin film for various characterizations.

## Results and Discussions

### Crystal Structure and X‐ray diffraction properties

**Figure 3:**
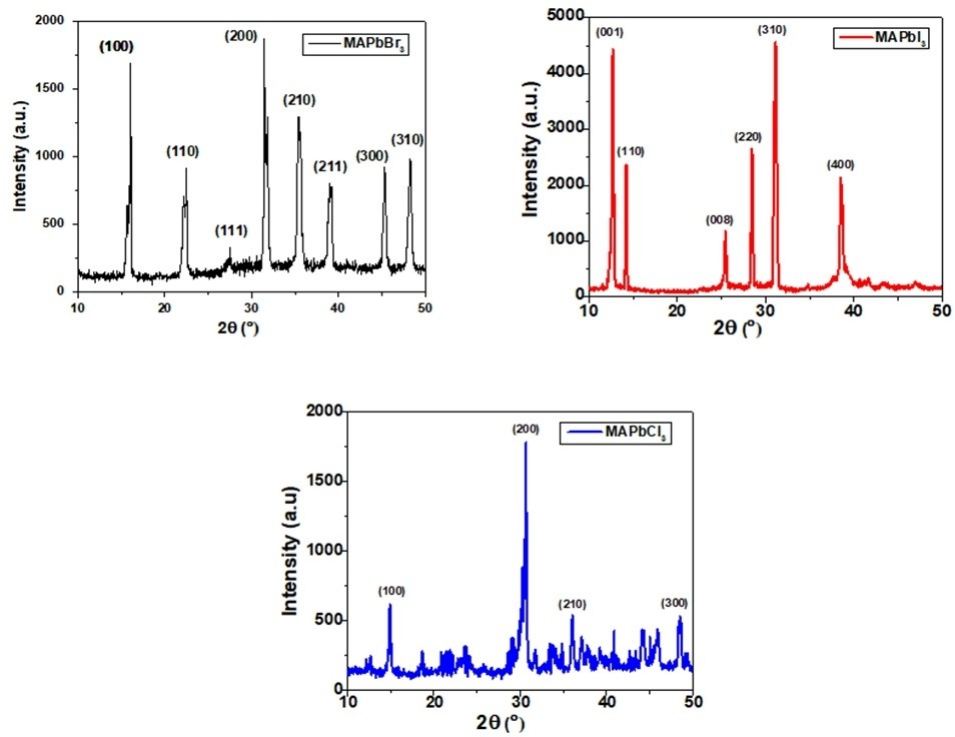
X‐ray diffraction patterns of MAPbX_3_ (X=Cl, Br and I) perovskite samples.

The precursors′ MAX, PbX_2_ (X=Cl, Br, I) used in hot‐injection synthesis were of high purity and procured commercially from Sigma Aldrich. Figure 3 depicts the XRD pattern of MAPbX_3_ halide perovskites system, where this perovskite was mainly constituted of organic MAX and inorganic PbX_2_ (X=Cl, Br, I) materials. XRD patterns of the MAPbX_3_ perovskite reveal the improved crystalline structure of the metal halide perovskites. Here, the XRD spectra of MAPbBr_3_ show diffraction peaks at 2Ɵ values 14.8, 21.6, 27.5, 31.4, 35.4, 39.1, 45.3 and 48.3, corresponding to (100), (110), (111), (200), (210), (211), (300) and (310) lattice planes at room temperature respectively whereas MAPbI_3_ shows diffraction peaks at 2Ɵ values 12.7, 14.2, 25.5, 28.5, 31.1 and 38.5°, corresponding to (001), (110), (008), (220), (310) and (400) lattice planes respectively under normal conditions where the peak assigned to 12.7° shows the presence of few traces of undecomposed PbI_2_ even at high temperature. Although, the undecomposed PbX_2_ (X=Cl, Br) was not observed in the other halide perovskite. So, it can be inferred that the undecomposed PbI_2_ will not interfere with the optical properties of lead iodide perovskite. Generally, in MAPbI_3_ perovskite films, the dense layer of PbI_2_ only seems responsible for the larger grain size of perovskite films. Besides, this growth of perovskite films largely depended on the substrates′ surface energy, leading to improved device performance. However, the diffraction peaks of MAPbCl_3_ appeared at 2Ɵ values 14.9, 30.6, 36.0 and 48.6°, corresponding to (100), (200), (210) and (300) lattice planes, respectively. XRD spectra reveal that all the three significant peaks of halide perovskite MAPbX_3_ (X=Cl, Br, I) observed approximately at 2Ɵ values of 14.7, 30.8 and 35.2° in all the recorded XRD patterns were shifted towards higher 2Ɵ side from I to Br to Cl which indicates the decrease in lattice‐spacing parameters as expected from literature. The narrow peaks in the XRD patterns depict a slightly larger crystallite size of the perovskite samples.

The crystallite size and strain in perovskite samples were calculated using the Williamson‐Hall plot^[49]^ for different applications of perovskite in solar cells. Generally, the W‐H plot is attributed to the diffraction line or peak broadening according to the given Equation 1:

(1) βcosΘ=4ϵsinΘ+kλ/D

Where, β=Full width at half maxima (FWHM), K=shape factor, λ=Wavelength of the X‐ray source, Ɵ=Bragg's angle, D=Crystallite size, ϵ=Lattice strain.

**Figure 4:**
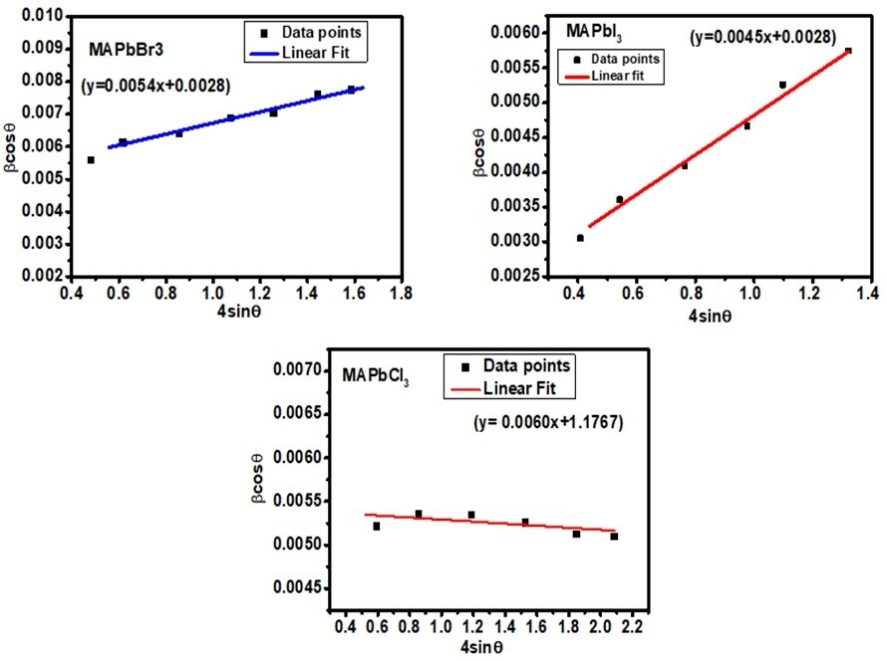
Variation of Williamson‐Hall plot for MAPbX_3_ perovskite samples.

Here, the β and Ɵ values were acquired from the XRD data. The W‐H graph was plotted, as shown in Figure 4, between 4sin Ɵ on the x‐axis and βcos Ɵ plotted on the y‐axis, which provides information about the line‐broadening. Depending on the graphical notation, the W‐H equation was compared with the straight‐line equation: y=mx+c. In comparison, the reciprocal of the intercept drawn on the x‐axis in the W‐H plot gives the average crystallite size, while the slope gives the lattice strain of the material.

The values obtained from the W‐H plot were compared with the size obtained from Debye Scherrer's equation. In the Debye‐Scherrer method,[^50^, ^51^] the crystallite size can be calculated based on the given Equation 2:

(2) D=kλ/βcosΘ

Where, β=Full width at half maximum (FWHM), K=Scherrer constant(0.9), λ=Wavelength of X‐ray source (λ=1.5406 Å), Ɵ=Bragg's angle (taken for most intense peak), D=Crystallite size.

The crystallite‐size obtained from the Debye Scherrer equation is 38.75 nm, 32.55 nm and 28.72 nm, corresponding to MAPbI_3_, MAPbBr_3_ and MAPbCl_3_, respectively. While crystallite size obtained for MAPbI_3_, MAPbBr_3_, and MAPbCl_3_ perovskite using the W‐H plot is 34.36 nm, 28.63 nm and 25.77 nm, respectively. As reported, the increase in crystallite size leads to reduced crystal defects, a lowering in trap‐state density and an increase in electron lifetime. However, lattice strain usually appears in lattice constants due to the imperfections in crystal, and the lattice strain was calculated to be 0.0028, 0.0017 and 1.1767, corresponding to MAPbBr_3_, MAPbI_3_ and MAPbCl_3_, respectively. The lower strain value leads to lower crystal structure distortion, effectively showing the lesser imperfection in the lattice and crystal defects. The crystallite size value obtained from the W‐H plot and Debye‐Scherrer equation is in close proximity with each other and the literature. Table 2 shows all the structural parameters obtained experimentally for the halide perovskite samples.

**Table 2:** Structural parameters of MAPbX_3_ perovskites.

Sample Name	Crystallite size (nm)		Particle size–TEM (nm)	Strain	Phase	Lattice Parameters (Å)			Volume (Å^3^)	Density (g/cm^3^)
	W‐H plot	Scherrer				a	b	c		
MAPbI_3_	34.36	38.75	44.5	0.0017	Tetragonal	8.88	8.88	9.94	783.81	5.25
MAPbBr_3_	28.63	32.55	38.8	0.0028	Cubic	5.75	5.75	5.75	190.11	33.47
MAPbCl_3_	25.77	28.72	31.1	1.1767	Cubic	5.76	5.76	5.76	190.10	24.15

Moreover, the MAPbX_3_ perovskites crystallize in different phases at room temperature, as MAPbI_3_ crystallize in the tetragonal phase[^52^, ^53^, ^54^] whereas MAPbCl_3_ and MAPbBr_3_ crystallize in the cubic phase.[^55^, ^56^] For MAPbBr_3_ and MAPbCl_3_, the lattice constants were calculated as a=b=c=5.75 Å and 5.76 Å, respectively.^[52]^ These values of the lattice parameter are in very close agreement with the reported value.[^57^, ^58^] Here, the unit‐cell volume of perovskite samples is calculated using the given Equation 3:
(3)

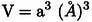




Where a is the lattice parameter of the MAPbBr_3_ and MAPbCl_3_ perovskite samples. And the X‐ray density (ρ) was calculated using the following Equation 4:

(4) $$\rho =\frac{8M}{N_{A}a^{3}}$$

where M=molecular weight of perovskite sample, a=lattice parameter, N_A_=Avogadro constant.

Using the above equations, the volume and density were calculated to be 190.11 Å^3,^ and 33.47 g/cm^3^ for MAPbBr_3_ and 190.10 Å^3^ and 24.15 g/cm^3^ for MAPbCl_3_, respectively, and these values are in good correlation with the reported literature. Whereas MAPbI_3_ crystallize in the tetragonal phase, and its lattice parameters possess the relation: a=b ≠ c, and these parameters were calculated to be a=b=8.88 Å and c=9.94 Å. Using the relation between volume and lattice parameters, V=a^2^c, the volume for MAPbI_3_ was calculated to be 783.81 Å^3^. The density of MAPbI_3_ perovskite can be calculated using the Equation (5):

(5) $$\rho =\frac{4M}{ca^{2}N_{A}}$$

and was calculated to be 5.25 g/cm^3^, which is in agreement with the literature values of lattice parameters and density of MAPbI_3_ perovskite samples crystallized in the tetragonal phase.

### Optical and Photoluminescence Spectra

**Figure 5:**
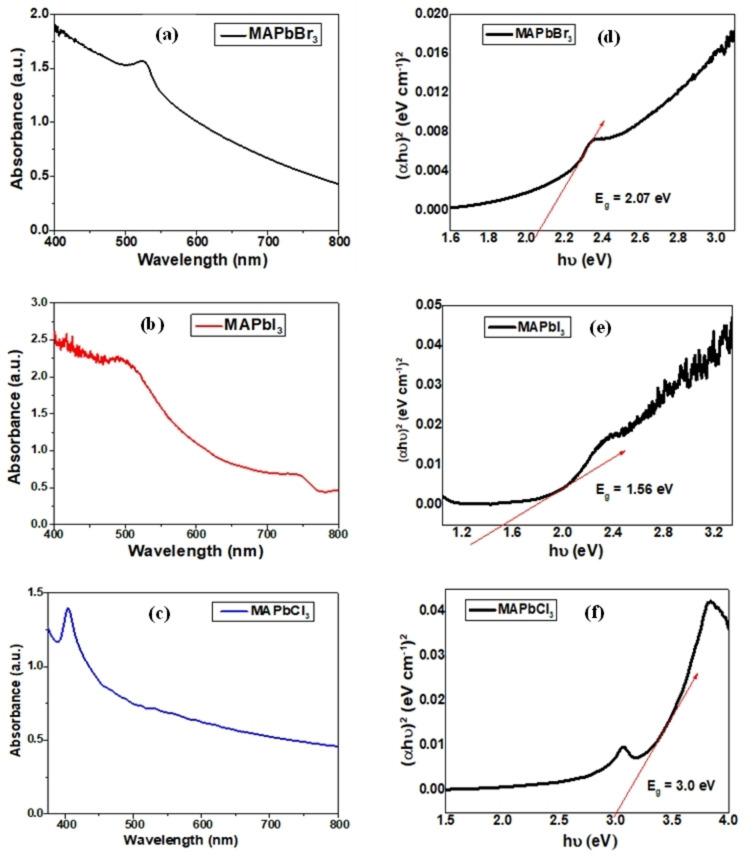
UV‐Visible spectra and band gap calculation using Tauc's plot for (a/d) MAPbBr_3_, (b/e) MAPbI_3_, and (c/f) MAPbCl_3_ perovskites.

Perovskite materials are known for their unique optoelectronic properties, enhanced photoluminescence (PL) characteristics, long carrier lifetime, and strong absorption, making them a promising candidate for solar cells. However, the band gap plays a vital role as it correlates well with the electronegativity difference between the cation and anion of perovskite materials. Herein, with an increase in perovskites band gap, the electronegativity of halide ion increases, which consequently reduces the covalent character of Pb^2+^‐halogen bonding.^[59]^ Thus, among halide ions, Cl^−^ ion is the most electronegative, which implies that MAPbCl_3_ possess the highest electronegativity difference between cation and anion as compared to other lead halide perovskites. It also leads to the weaker binding between Pb^2+^ and Cl^−^ and the highest band gap energy in MAPbCl_3_ perovskite. It can also be conferred that a decrease in the atomic weight of halide ion shifts the absorption band‐edge to the lower wavelength, resulting in the higher band gap of the perovskites. This statement is in good correlation with the experimental data, which depicts that MAPbCl_3_ possess the highest band gap energy of 3 eV while MAPbBr_3_ and MAPbI_3_ show the 2.07 and 1.56 eV band gap, respectively, as shown in Figure 5 (a to c). Here, the optical band gap energy of the halide perovskites was calculated using Tauc's Plot. The band gap of the material is calculated using Equation 6:
(6)

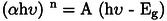




where, α **=**Absorption coefficient, E_g_=Band gap of the material, A=Constant, hυ = Energy of the incident photon (eV), n=nature of transition (n=2 and 1/2 for direct and indirect band gap respectively).

However, direct and indirect band gap differs based on the emission of a photon. As in the case of an indirect band gap, the photon was passed through the intermediate state and did not emit directly and also transfer the momentum of a crystal lattice, whereas, in the case of a direct band gap transition, the emission of a photon is direct due to the identical momentum of hole and electron in the conduction band and valence band.^[31]^

Although, the absorption coefficient (α) can be calculated using the Lambert‐Beer law:
(7)

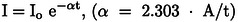




Where A=Absorbance of material, t=Thickness of the cuvette used in the analysis (1 cm).

Further, the graph was plotted between hυ on the x‐axis and (αhυ)^2^ on the y‐axis, and extrapolating the graph to obtain the band gap of the halide perovskite samples[^60^, ^61^] as shown in Figure 5 (d to f). Moreover, as the halogen ion composition varies Cl^−^ to Br^−^ to I^−^ in MAPbX_3_ perovskites, the absorption band‐edge shifts towards the higher wavelength region and lies in the 400–800 nm range. Therefore, the optical properties of perovskite material were affected with respect to the variation in halide composition. However, the results also inferred that the optical band gap varies with the different sizes of halide ions. Further, the iodide ion has the most significant size with the lowest band gap, while other halide perovskites with Br^−^ and Cl^−^ ions possess small atomic sizes, leading to increased optical band gap; even extrapolation creates the large iodine cavities for organic molecules. Figure 5 shows the UV‐Vvsible spectra along with the band gap calculation of the MAPbX_3_ perovskite sample.^[62]^ Generally, the band gap and size of the nanoparticles are inversely related to each other. Thus, the band gap increases with the reduced particle size or vice‐versa due to the proximity between the electron‐hole pair, which enhances their interaction and leads to higher energy. Therefore, the high band gap value depicts that high energy will be required to excite an electron from the valence band to the conduction band, leading to a higher frequency and low wavelength or blue shift in the absorption spectra. Thus, it can be concluded that a higher band gap or blue shift in absorption spectra in halide perovskite samples has promising applications in sensors, solar cells and photodetectors.^[63]^

**Figure 6:**
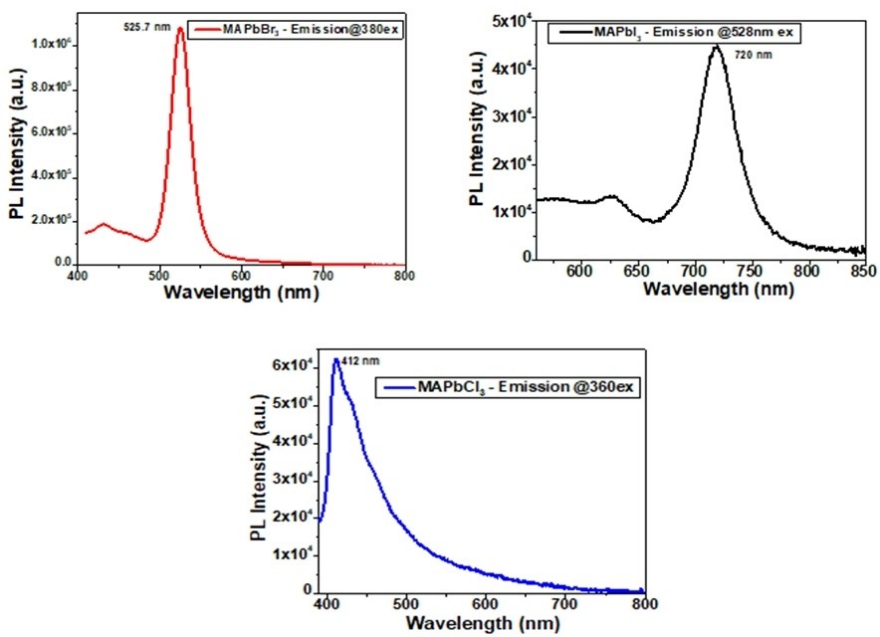
Photoluminescence spectra of MAPbX_3_ (X=I, Br, Cl) perovskite samples.

The lead halide perovskite shows a broad band of PL emission at room temperature. In all MAPbX_3_ (X=Cl, Br, I) perovskites, strong and broad PL emission was observed in the 400–800 nm range. PL spectra of MAPbI_3_ showbroad PL emission at 720 nm while narrow PL emission peak was observed at 525 nm and 412 nm corresponding to MAPbBr_3_ and MAPbCl_3_ perovskite, respectively, as shown in Figure 6. However, it was unusually observed that the PL emission of all the halide perovskites was smaller compared to their absorption spectra, and this property is beneficial for solar cell devices. As the halide composition varies from I to Br to Cl, blue shift PL emission with a narrower PL peak was observed, leading to low trap density in the materials. Moreover, the PL emission peak was observed at different excitation wavelengths of 528 nm, 380 nm and 360 nm corresponding to MAPbI_3_, MAPbBr_3_ and MAPbCl_3_, respectively and this difference in excitation spectra was attributed to the inorganic part of the perovskite structure.

However, in lead halide perovskites, PL intensity was different for different halides due to structural distortion in the compounds. Thus, it can be inferred that the significant PL emission peak originated from the Pb inner band lies between the Pb (6p) and Pb (6s) bands. These PL peaks suggested an independent behaviour from halide ion orbital mixing.[^64^, ^65^] In the case of MAPbBr_3_ perovskites, the richness of halide ions at the surface of QDs ought to be responsible for surface states. Here, the abundance of Br atoms on the surface will connect with cations, inhibiting the trapping of excited carriers and high PL quantum efficiency. This process is called self‐passivation, similar to the internal passivation behaviour of traditional QDs with halide ions. On the other side, the Br‐rich surface also binds with MA to form PbBr_x_ analogues, with a large band gap of MAPbBr_3_.

Time‐resolved photoluminescence (TRPL) measurements were done using the 266 nm laser for excitation. It helps to determine the decay in perovskites concerning exposure time. Here, we have also calculated the average carrier lifetime using the given Equation (8) for decay time:

(8) $$\tau_{av}=\;\frac{a_{1}\;\tau_{1}^{2}+\;a_{2}\;\tau_{2}^{2}}{a_{1}\tau_{1\;}+\;a_{2}\;\tau_{2}}$$

**Figure 7:**
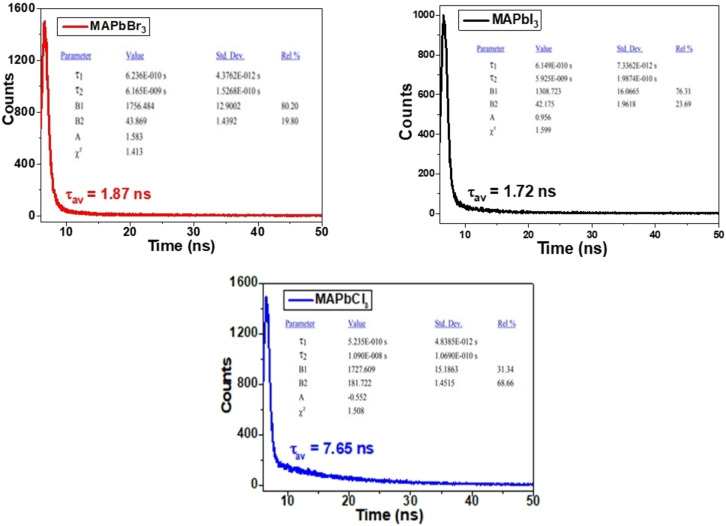
Time‐resolved photoluminescence spectra of MAPbX_3_ (X=Cl, Br, I) perovskite samples.

Where, a_i_=Amplitude of the i^th^ lifetime component, τ_i_=Respective lifetime value.

a_i_ and τ_i_ are fitting parameters described based on the fit results of the perovskite material. Using the above formula, the carrier lifetime was calculated to be 1.72 ns, 1.87 ns and 7.65 ns for MAPbI_3_, MAPbBr_3_ and MAPbCl_3_ perovskite, respectively, as shown in TRPL spectra in Figure 7. Hence, the decay lifetime follows the order MAPbCl_3_>MAPbBr_3_>MAPbI_3_ with .65 ns>1.87 ns>1.72 ns. As among all halide perovskites, MAPbCl_3_ possesses the highest average lifetime, leading to higher charge separation.

### SEM and TEM Study

#### FE‐SEM

**Figure 8:**
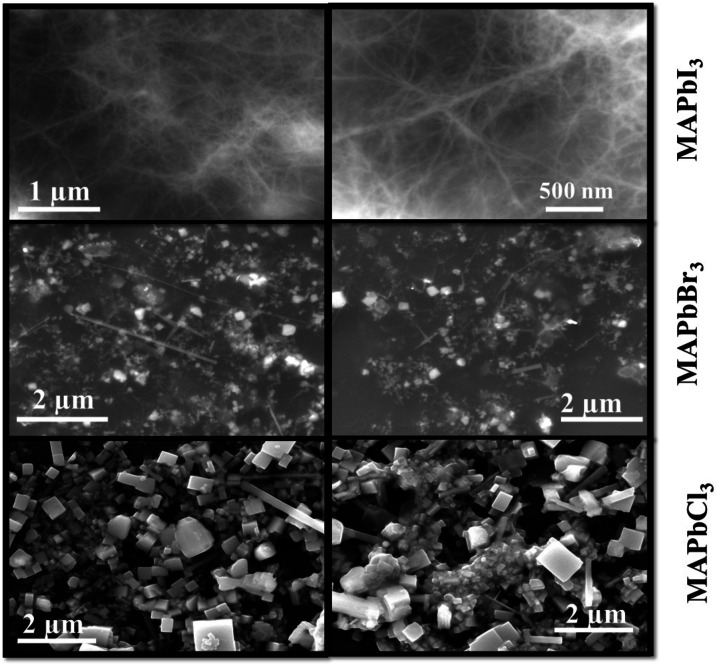
Surface morphology images of MAPbX_3_ (X=I, Cl, Br) perovskite samples.

Figure 8 demonstrates the surface morphology of the MAPbX_3_ (X=Cl, Br, I) perovskites recorded using 15 keV FE‐SEM. The FE‐SEM micrographs of MAPbI_3_ show an entangled structure of rods, MAPbBr_3_ shows the mixture of rods and spherical nanocrystals, while MAPbCl_3_ shows the presence of cubic nanocrystals. All the perovskites show very small agglomeration,that may also arise because of surface charge on the nanocrystals. The corresponding mean diameter observed from each SEM image lies between 30–40 nm, which agrees with the TEM data.

#### TEM

**Figure 9:**
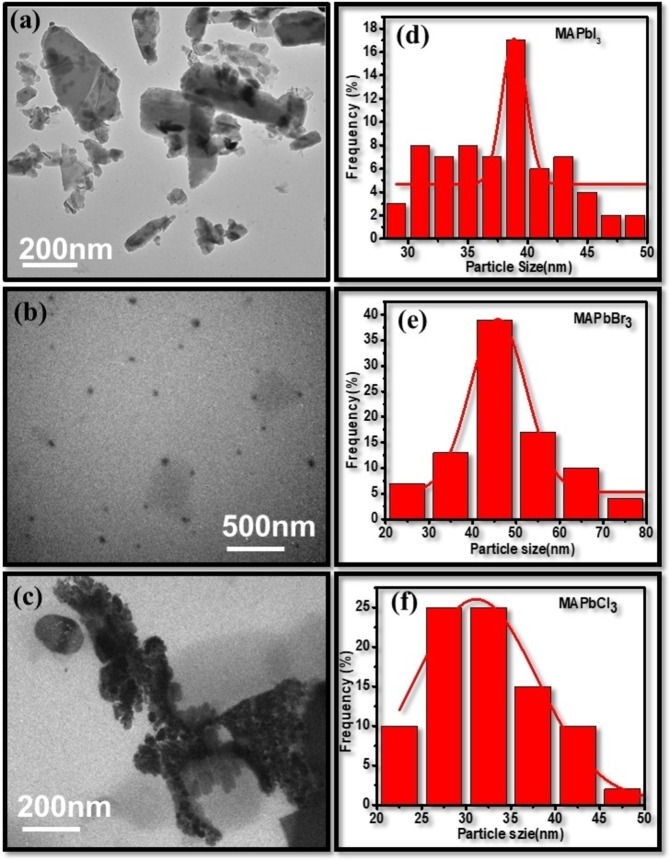
TEM images with particle size distribution (Gaussian distribution) for (a/d) MAPbI_3_, (b/e) MAPbBr_3_, and (c/f) MAPbCl_3_ perovskites.

The morphology and particle‐size distribution of MAPbX_3_ (X=Cl, Br, I) perovskites were recorded using TEM as shown in Figure 9. The TEM images of halide perovskites confirm the presence of spherical particles with their uniform distribution. The average size of particles was defined by selecting around 70–80 particles in each image. It was depicted in Figure 9 that particle size varies with halide variation, and size lies in the range of 30–45 nm for all the halide perovskites. As for MAPbI_3_, particle size was calculated to be 44.5 nm, 38.8 nm for MAPbBr_3_ and 31.1 nm for MAPbCl_3_. But it was noticed that the particle size of all halide perovskites lies in close proximity to the crystallite size calculated using W‐H plot in XRD data. The TEM histogram represents the particle size distribution (Gaussian distribution) of all the halide perovskites samples.

### Photovoltaic application through device simulations

**Figure 10:**
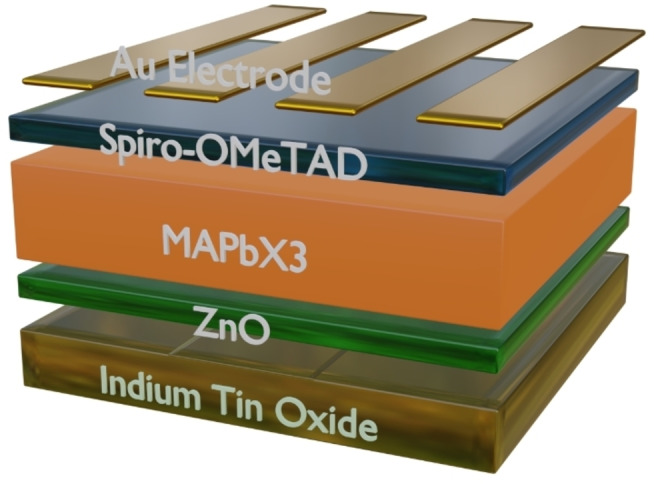
Schematic of MAPbX_3_‐based perovskite solar cells used during simulations.

After synthesizing and characterizing methylammonium halide‐based perovskites in terms of the structural, morphological, and optical properties using XRD, FESEM, TEM, UV‐Vis, PL and TRPL characterization techniques, the PV potential is investigated through SCAPS‐1D device simulations. SCAPS‐1D is developed at the University of Ghent, Belgium^[66]^ and is widely adopted to perform realistic device simulations of solar cells.[^67^, ^68^, ^69^, ^70^] Optoelectronic parameters obtained through experimental observations are supplied to perform the device simulations. Three different perovskite solar cells (PSCs) are simulated with different absorber layers, that is, MAPbI_3_, MAPbBr_3_ and MAPbCl_3_. A schematic of the device structure is shown in Figure 10. Spiro‐OMeTAD (460 nm) and ZnO (90 nm) are utilized for hole and electron transport layers, and their properties are obtained from the literature.[^71^, ^72^]

**Figure 11:**
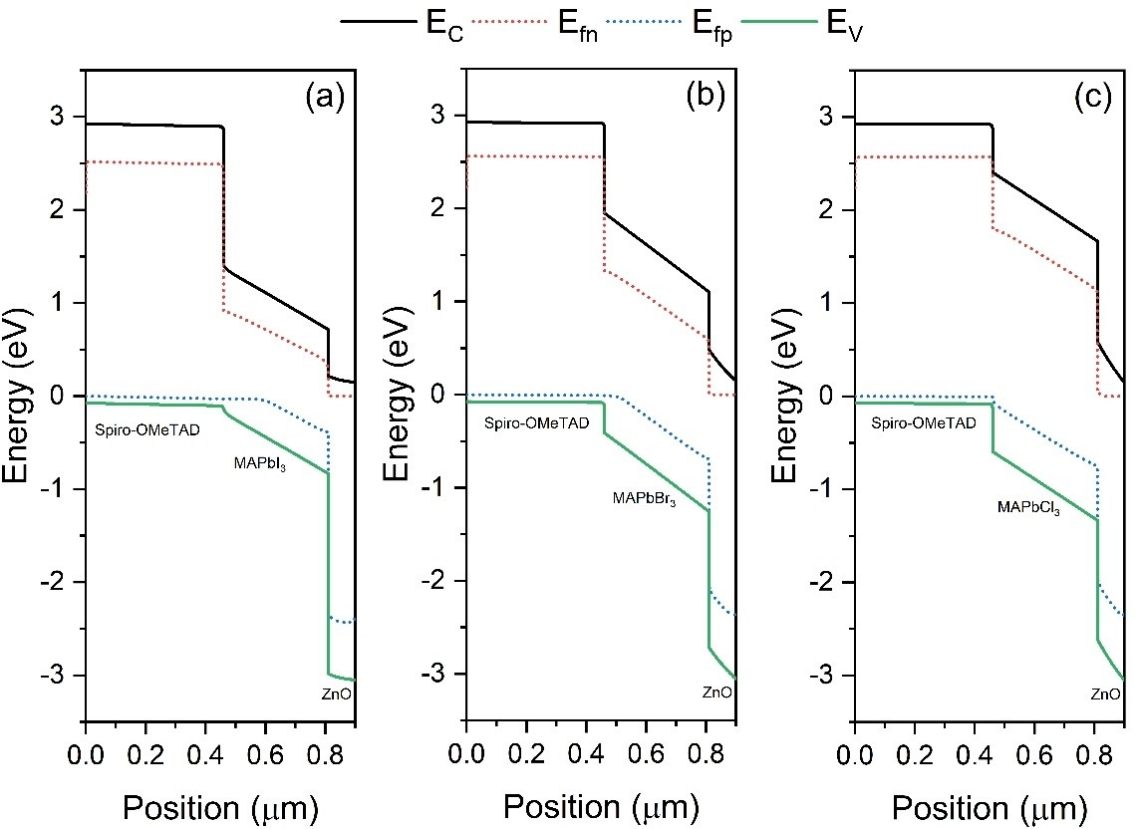
Energy band diagram under illumination for (a) MAPbI_3_, (b) MAPbBr_3_ and (c) MAPbCl_3_ based PSCs.

Energy band diagrams are obtained for all three devices and shown in Figure 11 (a–c) to understand the carrier dynamics within the devices. Upon illumination, quasi‐Fermi levels are formed, and electrostatic potential within the device results in the bending of bands. The associated electric field at absorber/HTL and absorber/ETL ensured the movement of light‐generated electrons towards ETL and holes towards HTL in all three devices. For the appropriate functioning of PSCs, the band offset of the absorber layer with the transport layer should be as minimal as possible. It has been observed that while replacing the absorber layer from MAPbI_3_ to MAPbCl_3_, a higher offset is observed with ETL and HTL layers. Therefore, a reduction in performance can be anticipated.

**Figure 12:**
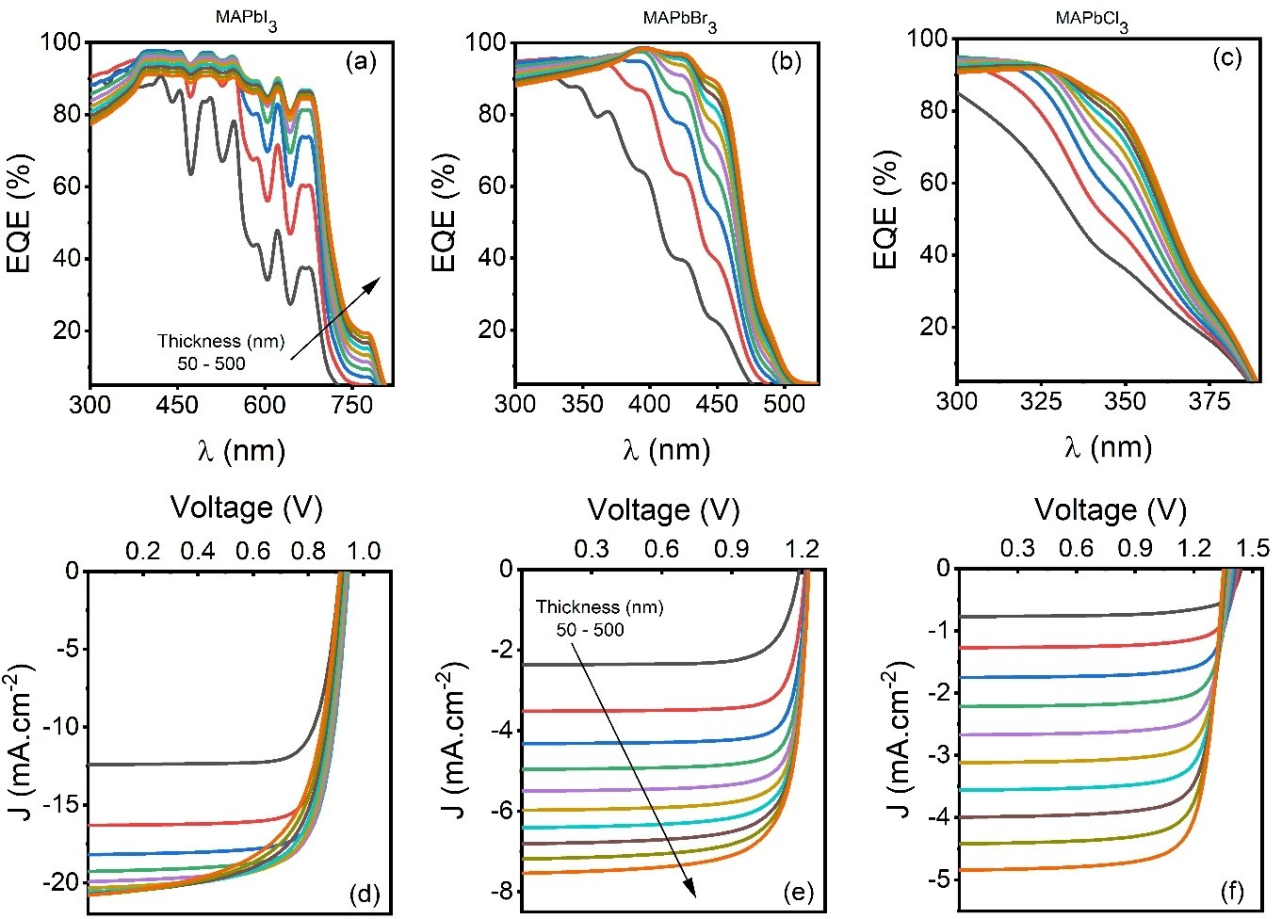
EQE and J‐V curves at different thicknesses for (a)/(d) MAPbI_3_, (b)/(e) MAPbBr_3_ and (c)/(f) MAPbCl_3_ based PSCs.

The amount of light‐generated carriers directly depends on the absorption coefficient and absorber layer thickness of the active‐layer; therefore, it is important to investigate the impact of active layer thickness on the PV performance of the devices.^[73]^ Therefore, optoelectronic performance in terms of external quantum efficiency (EQE) and illuminated current density‐voltage (J‐V) curve at varying thicknesses are obtained as shown in Figure 12(a–f) and studied to understand the PV potential of the synthesized perovskite materials. Increasing the thickness increases the absorption in the active layer and results in higher J_SC_ values; however, it is worth noting that carrier lifetime as obtained from TRPL showed the lowest carrier lifetime for MAPbI_3_; therefore, the recombination process also started at higher thicknesses due to lower diffusion length. This significantly reduces EQE, particularly at lower wavelengths than the other two materials. As band gap measured through Tauc's plot, they showed the lowest band gap for MAPbI_3_ and the highest for MAPbCl_3_; therefore, the cut‐off wavelength for EQE is higher for MAPbI_3_ than the other two perovskites. MAPbI_3_, owing to a lower band gap, ensures the superior harvesting of higher wavelength photons and results in higher J_SC_ values than the other two perovskites.

**Figure 13:**
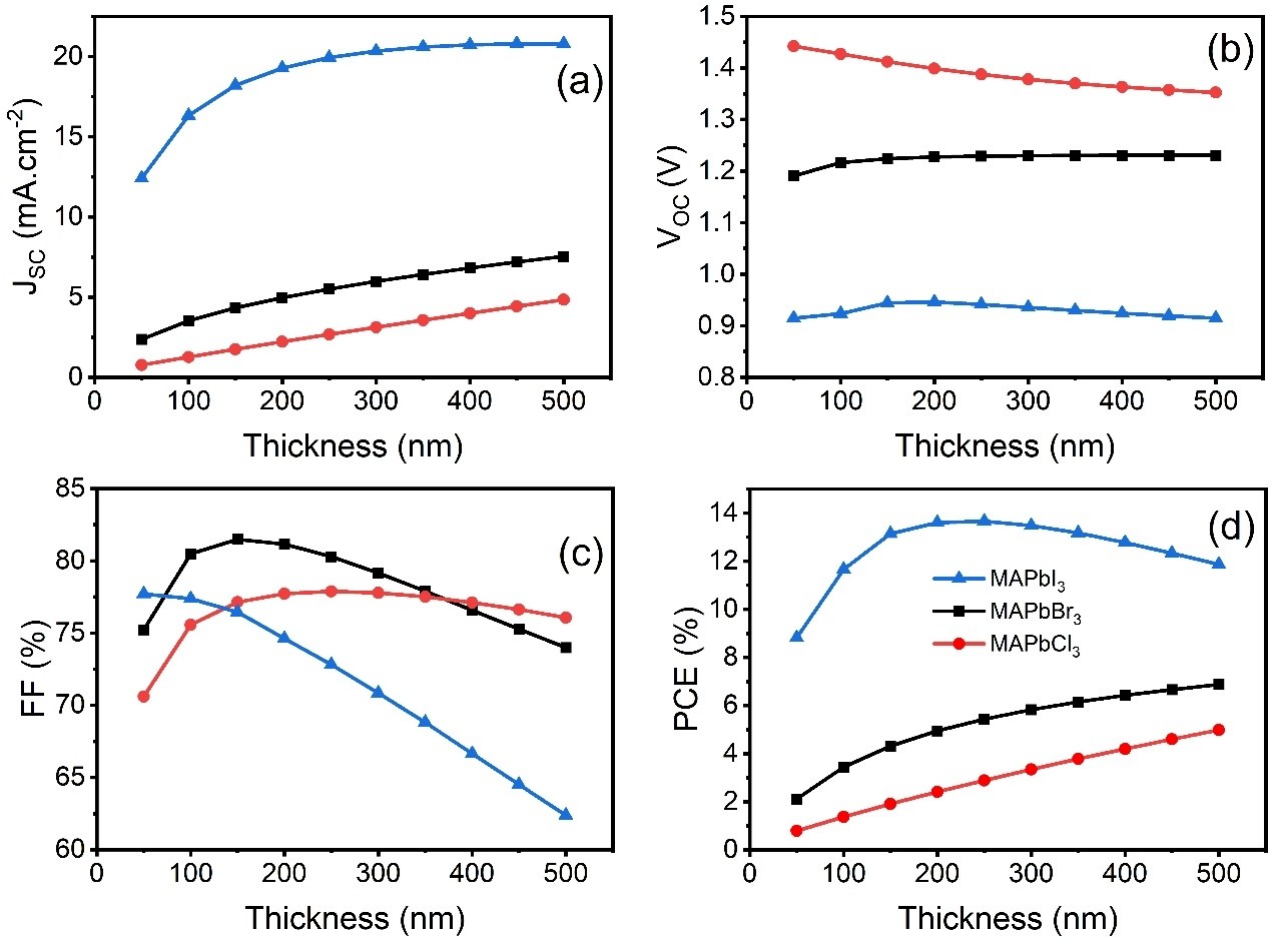
(a) J_SC_, (b) V_OC_, (c) FF, and (d) PCE curves at different thicknesses for MAPbI_3_, MAPbBr_3_ and MAPbCl_3_‐based PSCs.

PV parameters are also plotted as shown in Figure 13(a–d) to quantify the J_SC_, V_OC_, FF and PCE at different thicknesses for three different devices. Increasing the thickness increases the J_SC_ due to higher absorption and subsequent generation of charge carriers. However, MAPbI_3_ saturates after 250 nm thickness due to absorption saturation. V_OC_ remains constant or shows marginal reduction due to elevated recombination at higher thicknesses. FF showed an exciting trend. The MAPbI_3_ device showed a reduction in FF, whereas the other two devices showed improvement to a specific thickness, followed by reduction while increasing thickness beyond that point. Increasing the thickness increases the separation between collecting interfaces and reduces the strength of the electric field across the absorber layer but reduces the FF in MAPbBr_3_ and MAPbCl_3_ at lower thicknesses. Insufficient absorption resulted in lower J_SC_ and FF due to the low carrier generation rate, and increasing the thickness increases the absorption and FF, but after a certain point, reduction in strength of the electric field across the absorber layer started to dominate and reduced the FF.

The overall impact of J_SC_, V_OC_ and FF are summarised on PCE, which showed a maximum conversion efficiency of 13.66 % for MAPbI_3_ at 250 nm and 6.87 % and 4.98 % for MAPbBr_3_ and MAPbCl_3_ at 500 nm, respectively. PCE is also tabulated in Table 3 for better understanding.

**Table 3:** Photovoltaic parameters of PSCs having different absorber layers.

S. No.	Absorber	Thickness (nm)	J_SC_ (mA cm^−2^)	V_OC_ (V)	FF (%)	PCE (%)
1	MAPbI_3_	250	19.9	0.94	72.8	13.66
2	MAPbBr_3_	500	7.5	1.23	73.9	6.87
3	MAPbCl_3_	500	4.8	1.35	76.1	4.98

## Conclusions

This paper discussed the study of variation in structural, optical and morphological properties of halide perovskites synthesized by the Hot‐injection method. Here, all the basic properties of perovskites, such as nanocrystalline size, band gap, morphology, carrier lifetime, etc. were presented that alter the halide variation from I to Br to Cl in halide perovskites. Thus, this paper infers that the structural modification in perovskite lattice with the replacement of halide ions leads to variation in the properties of perovskite material for solar cell devices. Here, Perovskites were simulated with different absorber layers to determine the PV potential, and correspondingly, the optoelectronic properties of MAPbX_3_ (X=I/Br/Cl) perovskites were evaluated. Therefore, the experimental result confers that MAPbI_3_ perovskite possesses a low band gap and low carrier lifetime, which in conjunction with the simulation data, reckons that MAPbI_3_ possess the highest PCE of ~13.7 %, as compared to ~6.9 % and 5.0 % with Br or Cl‐based halides. Thus, it is considered the optimized and promising candidate for solar cell device applications. The findings of this work via the correlation between the experimental and simulation data for HIM‐synthesized MAPBX_3_ (X=I/Br/Cl) perovskites have been reported for the first time and could pave the way for the development of cost‐effective non‐vacuum‐based perovskite solar cells.

## Conflict of interest

The authors declare that they have no competing financial interest or personal relationships that could have appeared to influence the work reported in this paper.

1

## Data Availability

The data that support the findings of this study are available from the corresponding author upon reasonable request.
